# Impact of patient characteristics on the efficacy and safety of landiolol in patients with sepsis-related tachyarrhythmia: Subanalysis of the J-Land 3S randomised controlled study

**DOI:** 10.1016/j.eclinm.2020.100571

**Published:** 2020-10-13

**Authors:** Naoyuki Matsuda, Osamu Nishida, Takumi Taniguchi, Masaki Okajima, Hiroshi Morimatsu, Hiroshi Ogura, Yoshitsugu Yamada, Tetsuji Nagano, Akira Ichikawa, Yasuyuki Kakihana, J-Land 3S Study Group

**Affiliations:** a Department of Emergency & Critical Care Medicine, Nagoya University Graduate School of Medicine, Nagoya, Japan; b Department of Anesthesiology & Critical Care Medicine, Fujita Health University School of Medicine, Aichi, Japan; c Department of Anesthesiology & Intensive Care Medicine, Kanazawa University, Ishikawa, Japan; d Intensive Care Unit, Kanazawa University Hospital, Ishikawa, Japan; e Department of Anesthesiology and Resuscitology, Okayama University Graduate School of Medicine, Dentistry and Pharmaceutical Sciences, Okayama, Japan; f Department of Traumatology and Acute Critical Medicine, Osaka University Graduate School of Medicine, Osaka, Japan; g Department of Anesthesiology and Pain Relief Center, The University of Tokyo Hospital, Tokyo, Japan; h Clinical Development Planning, Ono Pharmaceutical Co., Ltd., Osaka, Japan; i Department of Emergency and Intensive Care Medicine, Kagoshima University Graduate School of Medical and Dental Sciences, Kagoshima, Japan

**Keywords:** Ultra-short-acting β1-selective antagonist, Heart rate, Mortality, Adverse events, Septic shock

## Abstract

**Background:**

The J-Land 3S trial demonstrated that landiolol is effective and tolerated for treating sepsis-related tachyarrhythmias. Patient characteristics (e.g. baseline heart rate [HR], type of tachyarrhythmia, and concomitant disorders) may impact the outcomes of landiolol therapy. We performed subanalyses of J-Land 3S to evaluate the impact of patient characteristics on the efficacy and safety of landiolol for treating sepsis-related tachyarrhythmia.

**Methods:**

Patients (≥20 years old; *N* = 151) hospitalised with sepsis at 54 participating hospitals in Japan with HR ≥100 beats/min for ≥10 min accompanied by diagnosis of tachyarrhythmia were randomised 1:1 to conventional sepsis therapy alone (control group) or conventional sepsis therapy plus landiolol (landiolol group). The efficacy and safety of landiolol were assessed in prespecified analyses of patients divided into subgroups by baseline characteristics and in post hoc, multivariate analyses with adjustment for age and HR at baseline.

**Findings:**

The percentage of patients with HR of 60–94 beats/min at 24 h after randomisation (primary endpoint) was greater in the landiolol group in most subgroups in univariate unadjusted analyses and in multivariate logistic regression. The incidence of new-onset arrhythmia by 168 h and mortality by 28 days were also lower in the landiolol group in most subgroups in univariate and multivariate Cox proportional hazards models. No subgroups showed a markedly higher incidence of adverse events in univariate or multivariate logistic regression analyses.

**Interpretation:**

These results of the J-Land 3S study suggest that the efficacy and safety of landiolol are generally unaffected by key patient characteristics.

**Funding:**

Ono Pharmaceutical Co., Ltd.

**Research in context**

**Evidence before this study**

Sepsis is a life-threatening organ dysfunction caused by an inappropriate response to infection, and is frequently associated with cardiovascular disorders, such as tachycardia. Reducing the heart rate (HR) to <95 beats/min soon after the onset of tachycardia can improve the prognosis of patients with sepsis-related tachyarrhythmias. Landiolol is an ultra-short-acting β1-selective antagonist that is already available for the management of atrial fibrillation and atrial flutter in critically ill patients, including those with cardiac dysfunction or renal failure. Preliminary evidence also suggested that landiolol may be effective for the management of sepsis-related tachyarrhythmias, a possibility that was evaluated in the phase II/III J-Land 3S study. The study demonstrated that landiolol resulted in significantly more patients with sepsis-related tachyarrhythmia achieving a heart rate of 60–94 beats/min at 24 h and significantly reduced the incidence of new-onset arrhythmia. Landiolol was also well tolerated in these patients. The prognosis of sepsis-related tachyarrhythmias may also be influenced by patient characteristics or other clinical events, like acute kidney injury. Therefore, evidence is needed on the efficacy and safety of landiolol in patients with such factors.

**Added value of this study**

Here, we performed subanalyses of the J-Land 3S study in order to evaluate the efficacy and safety of landiolol in patients divided into subgroups according to a variety of baseline clinical characteristics associated with poor prognosis of sepsis-related tachyarrhythmias. The results of prespecified univariate analyses as well as post hoc multivariate analyses with adjustment for age and heart rate at baseline indicate that landiolol demonstrated promising efficacy (measured in terms of the percentage of patients with HR of 60–94 beats/min at 24 h, percentage of patients with new-onset arrhythmia, and mortality by 28 days) in most subgroups of patients relative to the control group. In terms of safety, we observed no subgroups of landiolol-treated patients with a profound increase in the incidence of adverse events relative to the control group based on univariate and multivariate analyses.

**Implications of all the available evidence**

Results of the J-Land 3S study, as reported here and in the prior report, provide valuable evidence supporting the efficacy and safety of landiolol in patients with sepsis-related tachyarrhythmias. The present results also mimic the subanalyses performed in an earlier study (J-Land) in which patients with atrial fibrillation or atrial flutter complicated with left ventricular dysfunction were randomised to landiolol or digoxin. Those results, and the present findings, suggest that the efficacy and safety of landiolol is generally unaffected by baseline characteristics, supporting its use in a wide range of patients who develop sepsis-related tachyarrhythmias, for whom the prognosis is otherwise quite poor.

Alt-text: Unlabelled box

## 1 Introduction

Sepsis is defined as life-threatening organ dysfunction caused by a dysregulated host response to infection. Sepsis is known to induce sympathetic hyperactivity and exacerbate the inflammatory response accompanying infection, resulting in further organ damage and dysfunction [1], [2], [3], [4]. Although acute renal injury and acute respiratory distress syndrome (ARDS) are the predominant complications, many patients may experience septic shock due to excessive vasodilation and cardiac dysfunction [5], [6], [7]. A decrease in tissue circulation often causes a rise in lactic acid levels and metabolic acidosis.

Tachyarrhythmias (atrial fibrillation, atrial flutter, and sinus tachycardia) often develop in patients with sepsis due to excessive sympathetic hyperactivity and elevated levels of inflammatory cytokines. The onset of tachycardia or atrial fibrillation is an independent prognostic factor in patients with sepsis or other serious disorders [8], [9], [10], [11], [12], [13], [14], [15]. Tachyarrhythmias in critically ill patients are typically treated with β-blockers, calcium channel blockers, digitalis preparations, sodium channel blockers, or potassium channel blockers. However, these drugs may be contraindicated or of limited benefit in patients with cardiac dysfunction, renal failure, or other comorbidities (cardiogenic shock or acidosis, for example). Furthermore, the pharmacokinetic characteristics of these drugs may make them difficult to use in critically ill patients [16,17]. Accordingly, alternative drugs that can be used over a wide spectrum of conditions, regardless of comorbidities like cardiac dysfunction, renal failure, or acidosis, are needed for the treatment of sepsis-related tachyarrhythmias.

Landiolol is an ultra-short-acting β1-selective antagonist that is already available for the management of atrial fibrillation and atrial flutter in critically ill patients, including those with cardiac dysfunction or renal failure [18], [19], [20]. It is also used to treat intraoperative/postoperative tachyarrhythmias and ventricular fibrillation or ventricular tachycardia [18,21,22]. Preliminary evidence also revealed the potential use of landiolol for the management of sepsis-related tachyarrhythmias [23], [24], [25], a possibility that was evaluated in the J-Land 3S study [26]. This was a multicentre, open-label, phase II/III study performed in Japan in which patients who developed sepsis-related tachyarrhythmias (atrial fibrillation, atrial flutter, and sinus tachycardia) were randomised to receive conventional sepsis therapy with or without landiolol. The study showed that administration of landiolol was superior to conventional sepsis therapy in terms of lowering heart rate (HR) within 24 h after randomisation, with a lower rate of new-onset arrhythmia by 168 h after randomisation. The study showed that landiolol was also well tolerated in patients with sepsis-related tachyarrhythmias.

The type of tachyarrhythmia, the presence of other complications (e.g. acute kidney injury, ARDS), and other clinical characteristics may impact on the outcomes of sepsis-related tachyarrhythmia. Therefore, we performed prespecified and post hoc subanalyses of the J-Land 3S study in order to evaluate the efficacy and safety of landiolol in patients divided into subgroups according to these clinical characteristics.

## 2 Methods

The design of the J-Land 3S study is published in full elsewhere, together with its study protocol [26]. This study adhered to the Declaration of Helsinki and Good Clinical Practice, and was approved by the ethical review boards at all participating sites. This study was registered on the Japan Pharmaceutical Information Center - Clinical Trials Information database (JapicCTI-173767).

### 2.1 Patients

The inclusion and exclusion criteria are listed in more detail in our previous report [26]. Patients (≥20 years old) hospitalised at one of 54 hospitals in Japan who developed sepsis according to the Japanese Clinical Practice Guidelines for the Management of Sepsis and Septic Shock 2016 (J-SSCG 2016) [27] and the Third International Consensus Definitions for Sepsis and Septic Shock (Sepsis-3) [4] were eligible if their HR was maintained at ≥100 beats/min for ≥10 min without a change in catecholamine dose and was accompanied by a diagnosis of atrial fibrillation, atrial flutter, and/or sinus tachycardia. Only patients whose symptoms and signs could be confirmed within 24 h before randomisation and within 72 h after entering an intensive care unit could be registered. The attending physicians were required to stabilise the patient's hemodynamic status before randomisation. Written, informed consent was obtained from the patient or next of kin. Patients who subsequently developed a tachyarrhythmia and met the eligibility criteria were registered and eligible for randomisation [26].

### 2.2 Study design

Following enrolment, patients were randomised 1:1 to receive either conventional sepsis therapy alone (control group) or conventional sepsis therapy plus landiolol (landiolol group). Randomisation was stratified by HR at the time of randomisation (≥100 to <120 beats/min or ≥120 beats/min) and age (<70 years or ≥70 years). Patients in both groups were to be treated in accordance with the J-SSCG 2016 recommendations [27], including respiratory and fluid resuscitation, antimicrobials, and catecholamines, as deemed necessary. Prohibited and approved concomitant drugs are listed in the prior report [26].

### 2.3 Landiolol dosing

The administration of landiolol was mandatory for the first 96 h, starting within 2 h after randomisation. Its starting dose was 1 μg/kg/min (intravenous) and could be increased by 1 μg/kg/min generally every 15–20 min until the HR had decreased to <95 beats/min. Administration of landiolol was optional between 96 and 168 h, during which time the dose could be increased/decreased as appropriate by 1 μg/kg/min. The maximum permitted dose of landiolol was 20 μg/kg/min. After 96 h, the patient could be switched to oral or percutaneous β-blockers. The landiolol dose was to be reduced or discontinued if systolic blood pressure decreased by ≥20% from randomisation or if HR decreased to <60 beats/min.

### 2.4 Endpoints and analyses

The endpoints are listed in more detail in the prior report [26]. For the purpose of the present analyses, we focused on the primary endpoint (HR of 60–94 beats/min at 24 h after randomisation) and the following secondary/safety endpoints: new-onset arrhythmia by 168 h after randomisation, mortality by 28 days after randomisation, and adverse events by 168 h after randomisation.

Adverse events were defined as any undesirable or unintended sign (including abnormal laboratory values), symptom, or disease that occurred after randomisation, regardless of their causal relationship to the study. Exacerbation of an underlying disease or associated symptoms of the underlying disease, or complications that are medically judged to have gone beyond the extent of the natural course were also regarded as adverse events.

The four endpoints were compared between the landiolol and the control groups for subgroups of patients divided by baseline characteristics (see Table 1) that were considered likely to influence or confound the efficacy and safety outcomes. These analyses were prespecified in the statistical analysis plan and were conducted a priori.

**Table 1:** Patient characteristics.

	Overall	Landiolol group	Control group
	*N* = 151	*N* = 76	*N* = 75
Sex			
Male	90 (59⋅6)	52 (68⋅4)	38 (50⋅7)
Female	61 (40⋅4)	24 (31⋅6)	37 (49⋅3)
Age (years)			
<70	73 (48⋅3)	37 (48⋅7)	36 (48⋅0)
≥70	78 (51⋅7)	39 (51⋅3)	39 (52⋅0)
Mean (SD)	67⋅1 (14⋅5)	67⋅8 (13⋅8)	66⋅4 (15⋅2)
Heart rate (beats/min)			
<120	89 (60⋅1)	43 (58⋅9)	46 (61⋅3)
≥120	59 (39⋅9)	30 (41⋅1)	29 (38⋅7)
Missing	3	3	0
Mean (SD)	117⋅5 (14⋅0)	117⋅4 (14⋅7)	117⋅6 (13⋅4)
Diagnosis			
Atrial fibrillation	29 (19⋅2)	17 (22⋅4)	12 (16⋅0)
Sinus tachycardia	121 (80⋅1)	58 (76⋅3)	63 (84⋅0)
Atrial fibrillation and atrial flutter	1 (0⋅7)	1 (1⋅3)	0 (0⋅0)
Left ventricular ejection fraction (%)			
<50	53 (35⋅3)	28 (37⋅3)	25 (33⋅3)
≥50	97 (64⋅7)	47 (62⋅7)	50 (66⋅7)
Missing	1	1	0
Mean (SD)	55⋅13 (15⋅43)	54⋅01 (14⋅47)	56⋅24 (16⋅35)
Systolic blood pressure (mmHg)			
<120	84 (55⋅6)	40 (52⋅6)	44 (58⋅7)
≥120	67 (44⋅4)	36 (47⋅4)	31 (41⋅3)
Mean (SD)	119⋅3 (22⋅2)	121⋅1 (22⋅9)	117⋅4 (21⋅4)
Infection site			
Respiratory organ	45 (29⋅8)	22 (28⋅9)	23 (30⋅7)
Other organ	106 (70⋅2)	54 (71⋅1)	52 (69⋅3)
Comorbid septic shock			
Yes	137 (90⋅7)	69 (90⋅8)	68 (90⋅7)
No	14 (9⋅3)	7 (9⋅2)	7 (9⋅3)
Comorbid acute kidney injury			
Yes	98 (64⋅9)	44 (57⋅9)	54 (72⋅0)
No	53 (35⋅1)	32 (42⋅1)	21 (28⋅0)
Comorbid ARDS			
Yes	31 (20⋅5)	19 (25⋅0)	12 (16⋅0)
No	120 (79⋅5)	57 (75⋅0)	63 (84⋅0)
pH			
<7⋅35	47 (31⋅1)	28 (36⋅8)	19 (25⋅3)
≥7⋅35	104 (68⋅9)	48 (63⋅2)	56 (74⋅7)
Mean (SD)	7⋅383 (0⋅091)	7⋅370 (0⋅100)	7⋅395 (0⋅079)
SOFA score (total)			
<10	66 (43⋅7)	33 (43⋅4)	33 (44⋅0)
≥10	85 (56⋅3)	43 (56⋅6)	42 (56⋅0)
Mean (SD)	10⋅1 (3⋅0)	10⋅0 (3⋅1)	10⋅1 (3⋅0)
APACHE II score			
<25	98 (64⋅9)	51 (67⋅1)	47 (62⋅7)
≥25	53 (35⋅1)	25 (32⋅9)	28 (37⋅3)
Mean (SD)	22⋅7 (8⋅7)	23⋅1 (8⋅9)	22⋅2 (8⋅6)
eGFR (mL/min/1⋅73 m^2^)			
<30	65 (43⋅0)	27 (35⋅5)	38 (50⋅7)
≥30	86 (57⋅0)	49 (64⋅5)	37 (49⋅3)
Mean (SD)	43⋅8 (31⋅1)	48⋅9 (32⋅4)	38⋅6 (29⋅0)
pH <7⋅4 and HCO_3_^–^ <24 mmol/L			
Yes mmol/L (See PDF proof attached at the beginning)"?>	63 (41⋅7)	32 (42⋅1)	31 (41⋅3)
No	88 (58⋅3)	44 (57⋅9)	44 (58⋅7)
pH <7⋅4 and PaCO_2_ >45 mmHg			
Yes	21 (13⋅9)	17 (22⋅4)	4 (5⋅3)
No	130 (86⋅1)	59 (77⋅6)	71 (94⋅7)

Values are *n* (%) or mean (SD). Analyses were done on an as-assigned basis (safety analysis set).

APACHE = Acute Physiology and Chronic Health Evaluation; ARDS = acute respiratory distress syndrome; beats/min = beats per minute; eGFR = estimated glomerular filtration rate; SD = standard deviation; SOFA = Sequential Organ Failure Assessment.

In the prespecified univariate analyses of patient subgroups, the percentage difference (landiolol minus control) with 95% Newcombe confidence intervals (CI) was determined for the percentages of patients with HR of 60–94 beats/min at 24 h after randomisation and the percentages of patients with adverse events by 168 h without adjustment for covariates. Unadjusted hazard ratios were determined using the Cox proportional hazards model (with 95% CI) for new-onset arrhythmia by 168 h after randomisation and mortality by 28 days after randomisation. As post hoc analyses, we used multivariate logistic regression to compare for the percentages of patients with HR of 60–94 beats/min at 24 h after randomisation and the percentages of patients with adverse events by 168 h, and the multivariate Cox proportional hazards model for new-onset arrhythmia by 168 h after randomisation and mortality by 28 days after randomisation among the prespecified subgroups of patients. The post hoc multivariate analyses were adjusted for age and HR at baseline, which were used as allocation factors for randomising patients to the study groups.

Landiolol dosing characteristics (maximum dose within 24 h, dose at 24 h, average dose during the study, maximum dose during the study, and total dosing time) were determined in each subgroup in a post hoc manner. Landiolol doses are reported as the mean (standard deviation).

As the analyses were conducted in an exploratory manner, no p-values were calculated. All analyses were performed using SAS version 9.3 (SAS Institute, Cary, NC, USA).

### 2.5 Role of the funding source

Employees of Ono Pharmaceutical Co., Ltd. contributed to study design, data analysis, and writing the manuscript. The corresponding author had full access to the data and was responsible for the decision to submit the manuscript.

## 3 Results

### 3.1 Patients

**Fig. 1:**
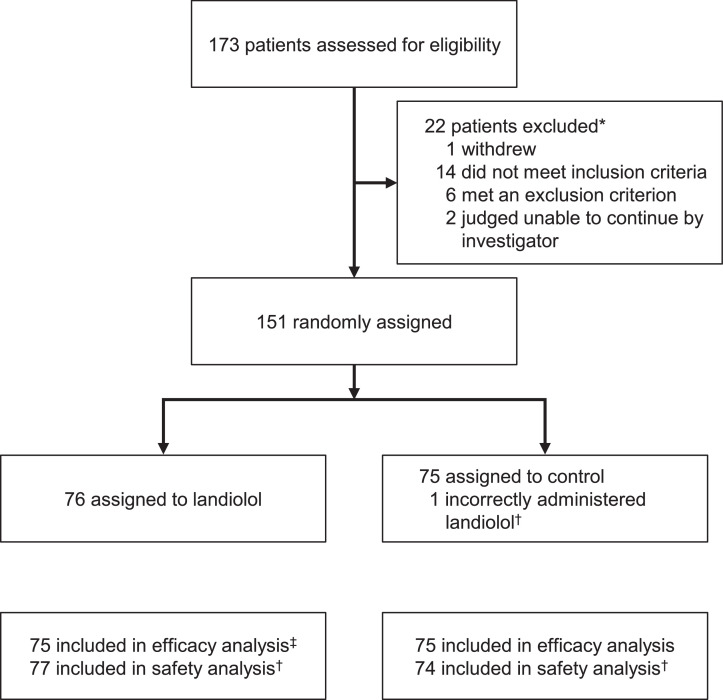
Trial profile (reprinted with permission from Kakihana et al. Lancet Respiratory Medicine 2020;8:863–872). *Multiple reasons may apply. ^†^One patient assigned to the control group was incorrectly given landiolol. This patient was included in the control group for the efficacy analysis and in the landiolol group for the safety analysis. ^‡^One patient assigned to the landiolol group did not meet the inclusion criteria but was administered landiolol. This patient was excluded from the efficacy analysis but was included in the safety analysis.

One hundred and fifty-one patients were randomised, of which 76 were allocated to the landiolol group and 75 to the control group (Fig. 1). The efficacy analysis set involved 75 patients in each group and the safety analysis set comprised 77 patients in the landiolol group and 74 in the control group. The baseline characteristics of patients are reported in Table 1. Both groups were generally well matched, except for the proportion of males, presence of comorbid ARDS, pH <7⋅35, and the combination of pH <7⋅4 and PaCO_2_ >45 mmHg, which were more frequent in the landiolol group, and the presence of comorbid acute kidney injury and eGFR <30 mL/min/1⋅73 m^2^, which were more frequent in the control group.

### 3.2 HR control

**Fig. 2:**
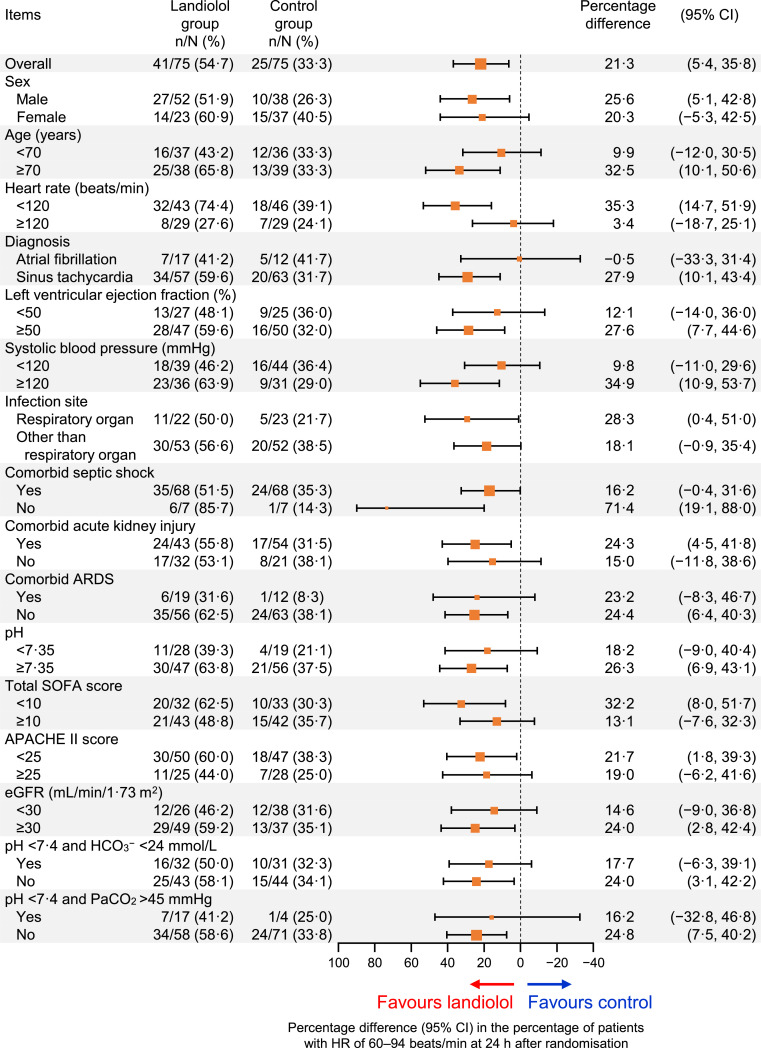
Subgroup analysis of the percentage of patients whose heart rate (HR) was adjusted to 60–94 beats/min at 24 h after randomisation. The size of the markers represents the number of patients included in the subgroup. Results of the multivariate analysis with adjustment for age and HR at baseline are shown in Fig. S1. APACHE = Acute Physiology and Chronic Health Evaluation; ARDS = acute respiratory distress syndrome; beats/min = beats per minute; CI = confidence interval; eGFR = estimated glomerular filtration rate; SOFA = Sequential Organ Failure Assessment.

As previously reported, the percentage of patients with HR 60–94 beats/min at 24 h was higher in the landiolol group than in the control group (54⋅7% [41/75] vs. 33⋅3% [25/75]) with a percentage difference of 21⋅3 (95% CI 5⋅4 to 35⋅8) in the univariate analysis (Fig. 2). This difference was also maintained in the multivariate analysis with adjustment for age and HR at baseline (odds ratio 2⋅80, 95% CI 1⋅37 to 5⋅69; Fig. S1). In univariate, unadjusted analyses (Fig. 2), the percentage of patients with HR 60–94 beats/min at 24 h was greater in the landiolol group in most subgroups of patients, and the numerically largest difference (landiolol−control) was found in patients without septic shock (percentage difference 71⋅4; 95% CI 19⋅1 to 88⋅0). Similar findings were also apparent in the multivariate analyses with adjustment for age and HR at baseline, and the largest difference (landiolol–control) was found in patients with respiratory organ infection (odds ratio 16⋅51; 95% CI 2⋅06 to 132⋅09) (Fig. S1). However, the efficacy of landiolol was attenuated in some subgroups, including patients with a baseline HR of ≥120 beats/min (unadjusted percentage difference 3⋅4; 95% CI −18⋅7 to 25⋅1; Fig. 2; adjusted odds ratio 1⋅36; 95% CI 0⋅40 to 10⋅36; Fig. S1) and patients with a diagnosis of atrial fibrillation (unadjusted percentage difference −0⋅5; 95% CI −33⋅3 to 31⋅4; Fig. 2; adjusted odds ratio 1⋅25; 95% CI 0⋅25 to 6⋅22; Fig. S1). In the control group, two of the seven patients with a baseline HR of ≥120 beats/min, and three of the five patients with atrial fibrillation were administered prohibited concomitant β-blockers as rescue therapy within 24 h of randomisation. None of the other baseline characteristics had a marked influence on the HR reduction with landiolol.

### 3.3 New-onset arrhythmia

**Fig. 3:**
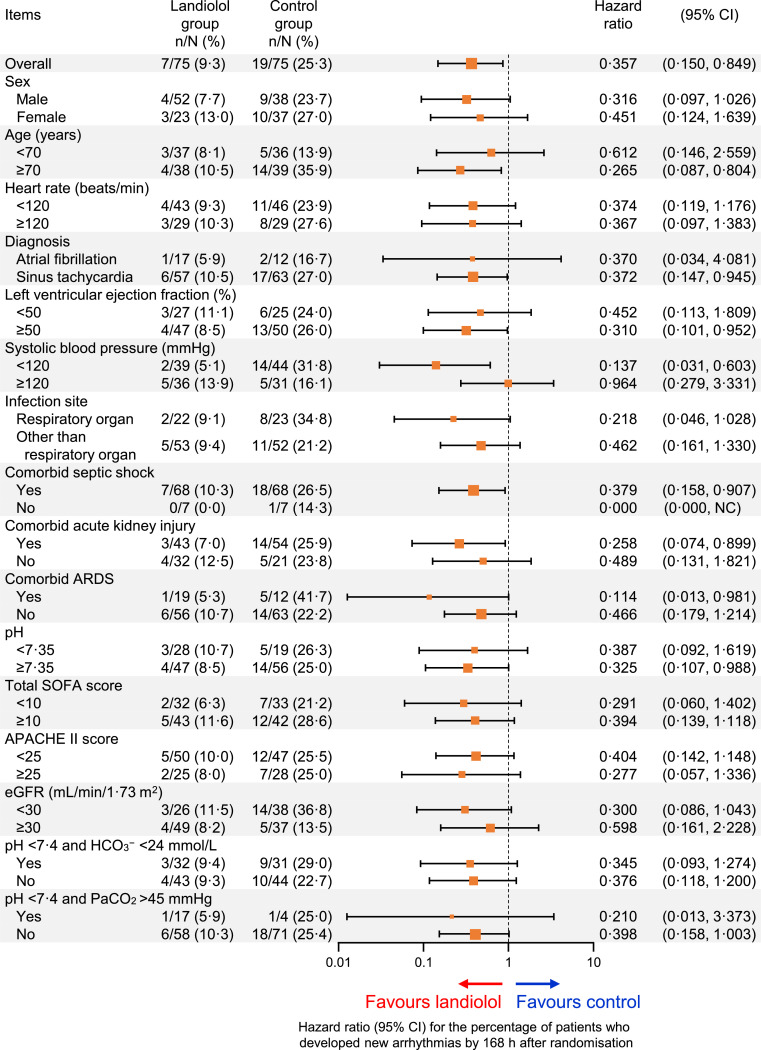
Subgroup analysis of the percentage of patients who developed new arrhythmias by 168 h after randomisation. The size of the markers represents the number of patients included in the subgroup. Hazard ratios are plotted on a log-scale. Results of the multivariate analysis with adjustment for age and heart rate at baseline are shown in Fig. S2. APACHE = Acute Physiology and Chronic Health Evaluation; ARDS = acute respiratory distress syndrome; beats/min = beats per minute; CI = confidence interval; eGFR = estimated glomerular filtration rate; NC = upper limit not calculable; SOFA = Sequential Organ Failure Assessment.

New-onset arrhythmia occurred by 168 h in 9⋅3% (7/75) of patients in the landiolol group versus 25⋅3% (19/75) of patients in the control group, with an unadjusted hazard ratio of 0⋅357 (95% CI 0⋅150 to 0⋅849) in favour of landiolol (Fig. 3). This difference was maintained after adjustment for age and HR at baseline with a hazard ratio of 0⋅351 (95% CI 0⋅147 to 0⋅835; Fig. S2). The unadjusted and adjusted hazard ratios for the incidence of new-onset arrhythmia generally favoured landiolol in most subgroups, except in patients with systolic blood pressure of ≥120 mmHg at baseline (unadjusted hazard ratio 0⋅964; 95% CI 0⋅279 to 3⋅331; Fig. 3; adjusted hazard ratio 0⋅997; 95% CI 0⋅276 to 3⋅607; Fig. S2).

### 3.4 Mortality by 28 days

**Fig. 4:**
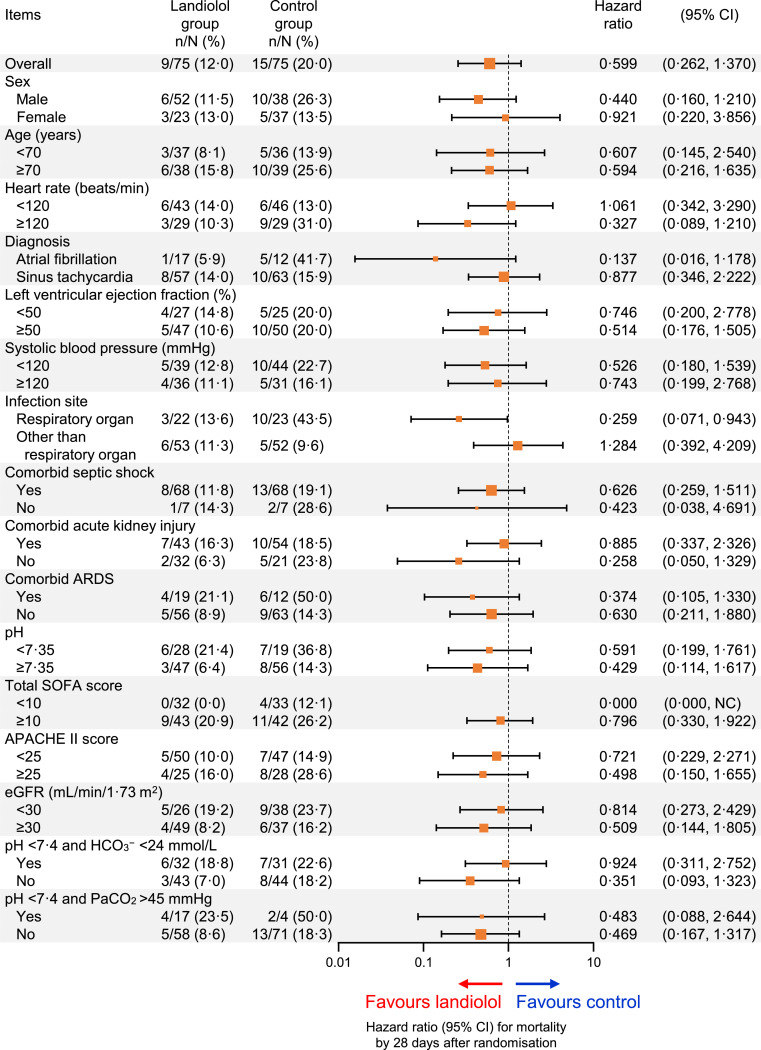
Subgroup analysis of mortality by 28 days after randomisation. The size of the markers represents the number of patients included in the subgroup. Hazard ratios are plotted on a log-scale. Results of the multivariate analysis with adjustment for age and heart rate at baseline are shown in Fig. S3. APACHE = Acute Physiology and Chronic Health Evaluation; ARDS = acute respiratory distress syndrome; beats/min = beats per minute; CI = confidence interval; eGFR = estimated glomerular filtration rate; NC = upper limit not calculable; SOFA = Sequential Organ Failure Assessment.

Nine of 75 patients (12⋅0%) in the landiolol group and 15 of 75 in the control group (20⋅0%) died by 28 days of randomisation with an unadjusted hazard ratio of 0⋅599 (95% CI 0⋅262 to 1⋅370; Fig. 4) and an adjusted hazard ratio of 0⋅598 (95% CI 0⋅261 to 1⋅366; Fig. S3), which tended to favour landiolol. The unadjusted and adjusted hazard ratios tended to favour landiolol in most subgroups of patients, although the 95% CIs crossed 1 for most comparisons (Figs. 4 and S3). This tendency was relatively strong among patients with a Sequential Organ Failure Assessment (SOFA) score of <10 (unadjusted and adjusted hazard ratio 0⋅000) or those with a respiratory organ infection (unadjusted hazard ratio 0⋅259; 95% CI 0⋅071 to 0⋅943, adjusted hazard ratio 0⋅188; 95% CI 0⋅040 to 0⋅893).

### 3.5 Adverse events

**Fig. 5:**
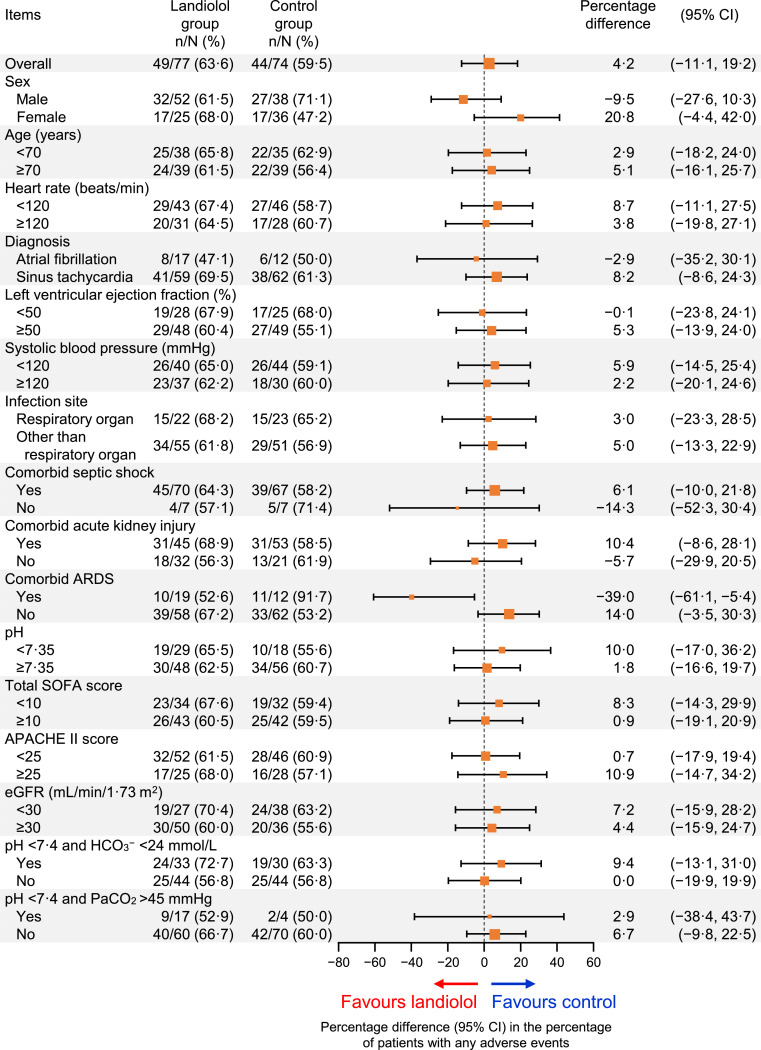
Subgroup analysis of overall adverse events. The size of the markers represents the number of patients included in the subgroup. Results of the multivariate analysis with adjustment for age and heart rate at baseline are shown in Fig. S4. APACHE = Acute Physiology and Chronic Health Evaluation; ARDS = acute respiratory distress syndrome; beats/min = beats per minute; CI = confidence interval; eGFR = estimated glomerular filtration rate; SOFA = Sequential Organ Failure Assessment.

Adverse events (of any grade or seriousness) occurred by 168 h in 63⋅6% (49/77) of patients in the landiolol group versus 59⋅5% (44/74) in the control group, with an unadjusted percentage difference of 4⋅2 (95% CI −11⋅1 to 19⋅2; Fig. 5) and adjusted odds ratio of 1⋅20 (95% CI 0⋅62 to 2⋅32; Fig. S4). Although the percentage of patients with adverse events was slightly greater with landiolol in most subgroups of patients, no subgroup showed a markedly higher incidence of adverse events in the unadjusted analyses (Fig. 5) or after adjustment for age and HR at baseline (Fig. S4). However, the percentage of patients with an adverse event was lower in the subgroup of patients with ARDS (52⋅6% [10/19] vs. 91⋅7% [11/12]; unadjusted percentage difference −39⋅0; 95% CI −61⋅1 to −5⋅4; adjusted odds ratio 0⋅09; 95% CI 0⋅01 to 0⋅92).

### 3.6 Landiolol dosing

The landiolol dose was analysed in patients stratified according to whether or not they experienced any of the four endpoints listed above, and in each of the patient subgroups. In all 76 patients who were allocated to landiolol, the maximum dose within 24 h was 6⋅11 (5⋅73) μg/kg/min, the dose at 24 h was 5⋅77 (5⋅57) μg/kg/min, the average dose during the study was 4⋅15 (4⋅35) μg/kg/min, and the maximum dose during the study was 6⋅96 (6⋅26) μg/kg/min. The mean duration of administration was 94⋅49 (43⋅49) h (Tables S1 and S2). When patients were stratified by the efficacy variables, we found that patients who achieved the primary endpoint, patients who did not experience new-onset arrhythmia, and patients who were alive at 28 days after randomisation appeared to have lower doses of landiolol than the other subgroups (Table S1). Landiolol doses also appeared to be lower in patients with adverse events than in patients without adverse events. When landiolol dosing was analysed according to patient subgroups (Table S2), there appeared to be differences in dosing characteristics among some subgroups. In particular, higher doses of landiolol were used in patients with HR ≥120 beats/min, patients with respiratory organ infection, and patients without comorbid septic shock. Two patients received the maximum permitted dose of landiolol at 24 h, and six patients at any time during the landiolol dosing period.

## 4 Discussion

The J-Land 3S study showed that the administration of landiolol is associated with a superior reduction in HR compared with conventional sepsis therapy, and is well tolerated in patients with sepsis-related tachyarrhythmias [26]. Because patients with sepsis-related tachyarrhythmias often present with clinically relevant comorbidities or conditions that may contraindicate or prevent the administration of other rate-controlling drugs, it is important to evaluate whether these characteristics may influence the efficacy or safety of landiolol. Therefore, we investigated the efficacy and safety of landiolol versus conventional sepsis therapy in patients divided into subgroups according to baseline characteristics. Overall, we found that landiolol was effective in the majority of subgroups of patients in terms of decreasing HR to 60–94 beats/min at 24 h, the rate of new-onset arrhythmia, and 28-day mortality, without marked increases in the incidence of adverse events. Nevertheless, some findings warrant discussion.

We found no clear efficacy advantage of landiolol in patients with a baseline HR of ≥120 beats/min and in patients with atrial fibrillation. Regarding baseline HR of ≥120 beats/min and atrial fibrillation, one explanation is that prohibited β-blockers were administered as rescue therapy within 24 h after randomisation to two of the seven patients in the control group with a HR ≥120 beats/min and to three of five patients with atrial fibrillation. This may contribute to a higher-than-expected rate of achieving the primary endpoint in the control group.

Another explanation for the lack of clear difference in some subgroups may relate to the landiolol dosing and caution about up-titrating the landiolol dose towards the maximum permitted dose, although the landiolol doses appeared to be greater in some subgroups of patients, including those who did not achieve HR control and patients with HR ≥120 beats/min at randomisation. The higher doses in the former subgroup may be due to a lower responsiveness to landiolol in individual patients, some of whom may have had a HR ≥120 beats/min at baseline. In some patients, a low dose may be sufficient to elicit the required HR lowering effect, but other patients, including those with higher baseline HR, might require higher landiolol doses to achieve adequate HR control. Because of the potential for excessive reductions in HR and blood pressure, it is recommended that landiolol administration is started at a low dose and its dose can be up-titrated based on the patient's HR response. However, the maximum permitted dose of landiolol (20 μg/kg/min) was not reached in most of the patients, which may indicate some reluctance to raise the landiolol dose towards the maximum. This may have limited the potential HR reduction in many patients, especially those with a high baseline HR. Perhaps counterintuitively, the dose of landiolol was higher in patients without any adverse events than in patients who experienced adverse events during the course of treatment. The reason for this is not clear, but it suggests that even high doses of landiolol are not necessarily associated with adverse events.

It is also possible that the clinicians were concerned about excessive reductions in HR with higher doses among patients with atrial fibrillation, but rescue therapy with another β-blocker was prohibited unless treatment was essential, and these agents were not administered to any patients in the landiolol group. Nevertheless, the total landiolol dosing time was about 20 h longer and the maximum landiolol dose was slightly higher in patients with atrial fibrillation than in patients with sinus tachycardia. It is possible that the landiolol dose was up-titrated more slowly in patients with atrial fibrillation, resulting in a lower reduction in HR after the 24 h period used for the primary endpoint.

The hazard ratios for mortality were generally low, in favour of landiolol, in patients with respiratory infection and in patients with a SOFA score of <10. Respiratory infections may progress to ARDS [28] and the mortality risk is higher in patients with ARDS complicated with new arrhythmia [29]. Landiolol may be associated with a reduction in new ARDS-associated arrhythmias in patients with respiratory infections. Meanwhile, it is thought that the pathophysiology of sepsis is milder in patients with lower SOFA scores, so the effect of landiolol on risk of death may be more apparent in these patients. In a prior study of esmolol, mortality by 28 days was 49⋅4% in the esmolol group versus 80⋅5% in the control group (adjusted hazard ratio 0⋅39; 95% CI 0⋅26 to 0⋅59) [30]. Their results and ours suggest a benefit of β-blockers on reducing mortality among patients with sepsis-related tachyarrhythmia. However, we should acknowledge the mortality rates were quite low in our study, and the results of these subanalyses should be interpreted carefully considering the small numbers of patients in each subgroup. Therefore, future studies may need to evaluate the impact of β-blockers on mortality among patients with sepsis-related tachyarrhythmias.

We also found marked differences in the percentages of patients with new-onset arrhythmias or adverse events between the landiolol and control groups among patients with ARDS. New-onset arrhythmia occurred in one of 19 patients in the landiolol group and in five of 12 patients in the control group, while adverse events occurred in ten of 19 and 11 of 12 patients, respectively. β-receptor stimulation has been reported to contribute to the induction of arrhythmia in ARDS [31]. Thus, we speculate that landiolol attenuated β-receptor stimulation, without increasing the risk of adverse events in patients with ARDS. β-blockers are generally contraindicated or administered with caution in patients with respiratory diseases because of the risk of bronchospasm [32]. It is interesting to note that esmolol was associated with an increase in stroke volume, maintenance of mean arterial pressure, and reduced the norepinephrine requirements in patients with septic shock [30], and a recent study revealed that administration of β-blockers improved oxygen saturation without affecting the mean arterial pressure or an increase in norepinephrine doses in patients on veno-venous extracorporeal membrane oxygenation [33]. Changes in hemodynamic factors were not assessed in our study, so future studies may need to evaluate the impact of landiolol on these factors in patients with septic shock and improve the prognosis of patients with ARDS. Nevertheless, it is reassuring to note that landiolol was efficacious and did not exacerbate adverse events in patients with ARDS in the present study.

The population of elderly people is steadily increasing in Japan, and it is expected that the incidence of sepsis and hence sepsis-related tachyarrhythmias will increase among older individuals not only in Japan but also worldwide [34], [35], [36]. Here, landiolol appeared to be more effective than conventional sepsis therapy in older patients, without markedly increasing the risk of adverse events. Thus, the present data suggest that landiolol is also suitable for use in older patients with sepsis-related tachyarrhythmias.

β-blockers, including landiolol, are generally contraindicated in patients with cardiogenic shock or acidosis (diabetic and metabolic) because the negative inotropic effects of β-blockade may exacerbate cardiac dysfunction in these settings [37]. However, many patients with sepsis develop septic shock or metabolic/respiratory acidosis. Shock may result from sepsis-induced myocardial dysfunction or reduced intravascular volume [27]. The present study included patients with shock and acidosis, and we observed consistent efficacy and safety of landiolol in patients with or without septic shock, in patients subdivided by baseline cardiac function (left ventricular ejection fraction [LVEF] ≥50% and <50%), and in patients with or without acidosis. These results suggest that landiolol can be used in patients with septic shock and metabolic or respiratory acidosis, under appropriate monitoring of HR and blood pressure.

Overall, our present results suggest that landiolol is safe and effective for treating sepsis-related tachycardia under HR and blood pressure monitoring, regardless of patient characteristics, such as septic shock, LVEF <50%, acidosis, comorbid acute renal injury, or severe sepsis.

Similar subanalyses were done in the J-Land study in which patients with atrial fibrillation or atrial flutter complicated with left ventricular dysfunction were randomised to landiolol or digoxin [19]. The analyses revealed better efficacy (percentage of patients with HR <110 beats/min at 2 h after start of intravenous infusion) of landiolol versus digoxin in a variety of subgroups of patients, including those with New York Heart Association class III, left ventricular ejection fraction of 25–35% or 35–50%, and by chronic kidney disease stage. Although the results of that study cannot be generalised to the clinical setting of the present study (or vice versa), the results of both studies suggest that the efficacy of landiolol is essentially unaffected by key baseline characteristics, and that the efficacy of landiolol may be more pronounced in certain subgroups of patients who may show poor responses to conventional therapies.

Some limitations of these analyses warrant mention. In particular, the overall sample size and numbers of patients in each subgroup were relatively small due to the study design, which did not require enrolling minimum numbers of patients in subgroups. Additionally, we did not perform statistical adjustments for multiplicity of analysis. These limitations may introduce some bias and should be considered when interpreting the results. Nevertheless, the subgroup analyses described here were performed in an exploratory manner and may support hypothesis-generation, but should not be considered as hypothesis-confirmatory. In the future, larger studies in certain high-risk groups of patients with sepsis-related tachyarrhythmias may help to confirm our findings. However, such studies may be impacted by a low accrual rate, especially for relatively rare conditions associated with sepsis/septic shock. In addition, we could not examine the efficacy or safety of landiolol in some high-risk patients, such as those with liver injury (e.g. total bilirubin ≥3 mg/dL), as these patients were excluded from the J-Land 3S study. Finally, this study was performed in Japan and enrolled Japanese patients, so the results may not be generalisable to other populations.

In conclusion, these exploratory results of the J-Land 3S study may suggest that the efficacy and safety of landiolol for the treatment of sepsis-related tachyarrhythmia are generally unaffected by most patient background characteristics. Future studies may provide more insight into the role of landiolol in this setting and help confirm our findings.

## Funding

This study was funded by Ono Pharmaceutical Co., Ltd.

## Contributions

NM and TN contributed to study conception and design. ON, TT, MO, HM, and HO contributed to study design and data acquisition. YY and AI contributed to study design. YK contributed to study conception and design, and data acquisition. All authors contributed to data interpretation; drafting, critical review, and final approval of the manuscript; and are accountable for the accuracy and integrity of the results. The corresponding author had full access to all the data in the study and had final responsibility for the decision to submit for publication.

## Data sharing statement

Qualified researchers may request Ono Pharma to disclose individual patient-level data from clinical studies through the following website: https://ClinicalStudyDataRequest.com. For more information on Ono Pharma's Policy for the Disclosure of Clinical Study Data, please see the following website: https://www.ono.co.jp/eng/rd/policy.html.

## Declaration of Competing Interest

NM reports consulting fees from Ono Pharmaceutical Co., Ltd. ON reports research grants and consulting fees from Ono Pharmaceutical Co., Ltd., and research grants from Fuso Pharmaceutical Industries, Ltd. and Asahi Kasei Pharma Corporation. MO, TT, HM, and HO report research grants and consulting fees from Ono Pharmaceutical Co., Ltd. YY reports consulting fees from Ono Pharmaceutical Co., Ltd. and research grants from Nihon Kohden Corporation. TN and AI are employees of Ono Pharmaceutical Co., Ltd. YK reports research grants and consulting fees from Ono Pharmaceutical Co., Ltd., and research grants from Japan Blood Products Organization and Asahi Kasei Pharma Corporation.

## Appendix Supplementary materials

Image, application 1

Image, application 2
