# Supplementary material for: Impact of patient characteristics on the efficacy and safety of landiolol in patients with sepsis-related tachyarrhythmia: Subanalysis of the J-Land 3S randomised controlled study
Source: eClinicalMedicine. 2020 Oct 13;28:100571. doi: 10.1016/j.eclinm.2020.100571 (PMC7700908; doi:10.1016/j.eclinm.2020.100571)

Appendix

Impact of Patient Characteristics on the Efficacy and Safety of Landiolol in Patients with Sepsis-Related Tachyarrhythmia: Subanalysis of the J-Land 3S Randomised Controlled Study

Naoyuki Matsuda MD, Osamu Nishida MD, Takumi Taniguchi MD, Masaki Okajima MD, Hiroshi Morimatsu MD, Hiroshi Ogura MD, Yoshitsugu Yamada MD, Tetsuji Nagano MSc, Akira Ichikawa MSc, and Yasuyuki Kakihana MD on behalf of the J-Land 3S Study Group

Contents **Page**

Contents	Page
List of investigators	1
Table S1. Landiolol doses according to the presence or absence of each endpoint (post hoc analysis)	2
Table S2. Subgroup analysis of landiolol doses (post hoc analysis)	3
Figure S1. Post hoc, multivariate subgroup analysis of the percentage of patients whose heart rate was adjusted to 60–94 beats/min at 24 h after randomisation	4
Figure S2. Post hoc, multivariate subgroup analysis of the percentage of patients who developed new arrhythmias by 168 h after randomisation	5
Figure S3. Post hoc, multivariate subgroup analysis of mortality by 28 days after randomisation	6
Figure S4. Post hoc, multivariate subgroup analysis of overall adverse events	7

J-Land 3S Study Investigators

Yoshiki Masuda, Sapporo Medical University Hospital; Shin Nunomiya, Jichi Medical University Hospital; Shingo Ihara, Nihon University Itabashi Hospital; Tetsuhiro Takei, Yokohama City Minato Red Cross Hospital; Osamu Nishida, Fujita Health University Hospital; Mitsuhiro Noborio, National Hospital Organization Osaka National Hospital; Hiroshi Morimatsu, Okayama University Hospital; Yasuyuki Kakihana, Kagoshima University Hospital; Satoshi Fujita, Asahikawa Medical University Hospital; Shigeki Kushimoto, Tohoku University Hospital; Masamitsu Sanui, Jichi Medical University Saitama Medical Center; Shigeto Oda, Taku Oshima, Chiba University Hospital; Hiroshi Tanaka, Juntendo University Urayasu Hospital; Hiroshi Morisaki, Keio University Hospital; Shinichi Ishimatsu, St. Luke's International Hospital; Nobutaka Chiba, Nihon University Hospital; Naoto Morimura, The University of Tokyo Hospital; Hidenobu Shigemitsu, Tokyo Medical and Dental University Hospital; Takaaki Murata, Shonan Kamakura General Hospital; Yasuyuki Tsujita, Shiga University of Medical Science Hospital; Hiroshi Ogura, Osaka University Hospital; Kyohei Miyamoto, Wakayama Medical University Hospital; Nobuaki Shime, Hiroshima University Hospital; Hiroshi Adachi, Iizuka Hospital; Mineji Hayakawa, Hokkaido University Hospital; Gaku Takahashi, Iwate Medical University Hospital; Akio Kimura, Center Hospital of the National Center for Global Health and Medicine; Takumi Taniguchi, Kanazawa University Hospital; Naoshi Takeyama, Aichi Medical University Hospital; Satoru Hashimoto, University Hospital Kyoto Prefectural University of Medicine; Satoshi Mizobuchi, Kobe University Hospital; Ryutaro Seo, Kobe City Medical Center General Hospital; Motohiro Sekino, Nagasaki University Hospital; Yuichi Kataoka, Kitasato University Hospital; Hideo Nishizawa, Yokohama Rosai Hospital; Hisakazu Kohata, Sakai City Medical Center; Gaku Inagawa, Yokohama Municipal Citizen's Hospital; Makoto Takatori, Hiroshima City Hiroshima Citizens Hospital; Hiroyasu Ishikura, Fukuoka University Hospital; Satoki Inoue, Nara Medical University Hospital; Yuichiro Sakamoto, Saga University Hospital; Yoshiaki Inoue, University of Tsukuba Hospital; Naoshige Harada, Japanese Red Cross Musashino Hospital; Hotaka Kumano, Higashiosaka City Medical Center; Kazuma Yamakawa, Osaka General Medical Center; Kensuke Nakamura, Hitachi General Hospital; Isao Tsuneyoshi, University of Miyazaki Hospital; Seiji Hitrota, Japanese Red Cross Kochi Hospital; Nobuya Kitamura, Kimitsu Chuo Hospital; Dai Miyazaki, Japanese Red Cross Maebasi Hospital; Yoshiro Hayashi, Kameda Medical Center; Tsuyoshi Ueno, Kagoshima City Hospital; Manabu Kakinohana, University of the Ryukyus Hospital; Shingo Adachi, Rinku General Medical Center; Akira Ichikawa, Keita Nagasawa, and Yuya Sakamoto, Ono Pharmaceutical Team.

Table S1. Landiolol doses according to the presence or absence of each endpoint (post hoc analysis)

Endpoint		n	Maximum dose within 24 h (µg/kg/min)	Dose at 24 h (µg/kg/min)	Average dose during the study (µg/kg/min)	Maximum dose during the study (µg/kg/min)	Total dosing time (h)
Overall		76	6·11 (5·73)	5·77 (5·57)	4·15 (4·35)	6·96 (6·26)	94·49 (43·49)
HR of 60–94 beats/min at 24 h (primary endpoint)*	Achieved	41	4·80 (4·43)	3·97 (3·79)	2·89 (2·54)	5·51 (5·00)	105·38 (30·25)
	Not achieved	34	7·64 (6·83)	8·57 (6·84)	5·72 (5·61)	8·59 (7·27)	80·58 (54·00)
New-onset arrhythmia by 168 h after randomisation*	Yes	7	7·39 (5·78)	11·21 (4·61)	5·27 (5·24)	8·54 (6·45)	58·23 (50·44)
	No	68	5·90 (5·77)	5·33 (5·46)	4·01 (4·31)	6·68 (6·25)	98·36 (41·62)
Died by 28 days after randomisation*	Yes	9	7·46 (6·38)	7·86 (5·16)	6·06 (5·17)	9·68 (7·20)	92·53 (68·41)
	No	66	5·85 (5·68)	5·43 (5·60)	3·86 (4·23)	6·46 (6·06)	94·78 (39·96)
Adverse events by 168 h after randomisation†	Yes	49	5·44 (4·91)	5·30 (5·07)	3·96 (4·22)	6·42 (5·83)	90·37 (45·63)
	No	28	7·44 (7·01)	6·58 (6·36)	4·53 (4·66)	8·00 (7·03)	102·57 (38·55)

Values are mean (standard deviation).
*Analyses were done on the efficacy analysis set.
†Analyses were done on the safety analysis set.
HR = heart rate

Table S2. Subgroup analysis of landiolol doses (post hoc analysis)

Baseline characteristic		n	Maximum dose within 24 h (µg/kg/min)	Dose at 24 h (µg/kg/min)	Average dose during the study (µg/kg/min)	Maximum dose during the study (µg/kg/min)	Total dosing time (h)
Overall		76	6·11 (5·73)	5·77 (5·57)	4·15 (4·35)	6·96 (6·26)	94·49 (43·49)
Sex	Male	52	6·06 (5·92)	5·73 (5·72)	3·89 (4·17)	6·70 (6·34)	94·72 (47·94)
	Female	24	6·22 (5·46)	5·86 (5·38)	4·70 (4·74)	7·49 (6·19)	94·01 (33·26)
Age (years)	<70	37	5·40 (5·43)	5·15 (4·77)	3·28 (3·32)	5·97 (6·02)	81·65 (45·45)
	≥70	39	6·79 (6·00)	6·28 (6·15)	4·98 (5·04)	7·89 (6·41)	106·65 (38·28)
Heart rate (beats/min)	<120	43	4·29 (3·88)	4·02 (4·04)	2·70 (2·41)	5·02 (4·83)	92·38 (40·80)
	≥120	30	8·85 (7·07)	8·44 (6·70)	6·37 (5·68)	9·88 (7·24)	97·53 (48·91)
Diagnosis	Atrial fibrillation	17	6·22 (5·96)	5·28 (6·03)	4·57 (5·06)	7·04 (6·22)	110·59 (45·37)
	Sinus tachycardia	58	5·84 (5·47)	5·64 (5·15)	3·80 (3·78)	6·71 (6·14)	89·80 (42·61)
Left ventricular ejection fraction	<50	28	6·72 (6·29)	6·59 (6·46)	4·75 (4·69)	7·81 (6·98)	95·31 (44·16)
(%)	≥50	47	5·85 (5·46)	5·30 (4·99)	3·85 (4·19)	6·56 (5·86)	96·04 (41·77)
Systolic blood pressure (mmHg)	<120	40	6·29 (5·81)	5·83 (5·68)	4·33 (4·40)	7·15 (6·43)	94·68 (43·74)
	≥120	36	5·90 (5·73)	5·70 (5·52)	3·95 (4·35)	6·72 (6·14)	94·26 (43·85)
Infection site	Respiratory organ	22	8·04 (6·95)	6·79 (6·84)	5·14 (5·60)	8·31 (7·13)	100·50 (38·12)
	Other organ	54	5·30 (4·99)	5·29 (4·86)	3·73 (3·69)	6·38 (5·83)	91·95 (45·68)
Comorbid septic shock	Yes	69	5·82 (5·66)	5·82 (5·85)	4·15 (4·51)	6·70 (6·27)	93·18 (44·87)
	No	7	8·96 (6·12)	5·41 (2·31)	4·20 (2·61)	9·39 (6·07)	106·98 (25·76)
Comorbid acute kidney injury	Yes	44	5·63 (5·08)	5·96 (5·27)	3·97 (4·16)	6·55 (5·84)	101·71 (44·94)
	No	32	6·75 (6·52)	5·53 (6·02)	4·39 (4·64)	7·49 (6·83)	85·01 (40·24)
Comorbid ARDS	Yes	19	6·37 (5·67)	6·86 (5·27)	5·24 (5·21)	7·39 (6·31)	87·52 (58·57)
	No	57	6·04 (5·80)	5·50 (5·66)	3·83 (4·06)	6·83 (6·29)	96·57 (38·29)
pH	<7.35	28	5·51 (5·59)	4·89 (5·14)	3·97 (4·46)	6·81 (6·60)	90·44 (51·06)
	≥7.35	48	6·46 (5·85)	6·22 (5·78)	4·26 (4·33)	7·04 (6·13)	96·81 (38·89)
Total SOFA score	<10	33	6·98 (6·20)	5·91 (5·87)	4·24 (3·98)	7·83 (6·60)	97·37 (35·76)
	≥10	43	5·42 (5·30)	5·65 (5·36)	4·08 (4·67)	6·26 (5·96)	92·17 (49·15)
APACHE II score	<25	51	6·08 (5·71)	5·39 (5·23)	3·58 (3·48)	6·59 (5·87)	86·40 (37·26)
	≥25	25	6·17 (5·90)	6·52 (6·23)	5·28 (5·60)	7·68 (7·02)	110·35 (50·79)
eGFR (mL/min/1·73 m^2)	<30	27	5·53 (4·95)	6·43 (5·41)	3·53 (3·89)	6·34 (5·79)	92·28 (60·19)
	≥30	49	6·45 (6·16)	5·49 (5·67)	4·51 (4·59)	7·31 (6·55)	95·76 (30·81)
pH <7·4 and HCO_3^- <24 mmol/L	Yes	32	4·73 (4·26)	4·40 (3·07)	3·25 (3·08)	5·76 (5·57)	79·37 (52·18)
	No	44	7·06 (6·43)	6·48 (6·40)	4·77 (4·98)	7·77 (6·63)	104·80 (33·26)
pH <7·4 and $PaCO_2$ >45 mmHg	Yes	17	6·85 (6·66)	5·56 (5·74)	4·78 (5·18)	8·16 (7·23)	107·99 (35·51)
	No	59	5·91 (5·50)	5·85 (5·57)	3·98 (4·13)	6·62 (5·99)	90·76 (45·00)

Values are mean (standard deviation). Analyses were done on an as-assigned basis (safety analysis set).
APACHE = Acute Physiology and Chronic Health Evaluation; ARDS = acute respiratory distress syndrome; eGFR = estimated glomerular filtration rate; SOFA = Sequential Organ Failure Assessment

Figure S1. Post hoc, multivariate subgroup analysis of the percentage of patients whose heart rate (HR) was adjusted to 60–94 beats/min at 24 h after randomisation using multivariate logistic regression with adjustment for age and heart rate

The size of the markers represents the number of patients included in the subgroup. Odds ratios are plotted using a log-scale. Results of univariate analyses by patient subgroups are shown in Fig. 2.

APACHE = Acute Physiology and Chronic Health Evaluation; ARDS = acute respiratory distress syndrome; beats/min = beats per minute; CI = confidence interval; eGFR = estimated glomerular filtration rate; SOFA = Sequential Organ Failure Assessment

Items	Landiolol group n/N (%)	Control group n/N (%)	Odds ratio	(95% CI)
Overall	41/75 (54·7)	25/75 (33·3)	2·80	(1·37, 5·69)
Sex				
Male	27/52 (51·9)	10/38 (26·3)	3·87	(1·40, 10·75)
Female	14/23 (60·9)	15/37 (40·5)	2·44	(0·82, 7·29)
Age (years)				
<70	16/37 (43·2)	12/36 (33·3)	2·03	(0·70, 5·88)
≥70	25/38 (65·8)	13/39 (33·3)	3·93	(1·49, 10·36)
Heart rate (beats/min)				
<120	32/43 (74·4)	18/46 (39·1)	4·49	(1·81, 11·11)
≥120	8/29 (27·6)	7/29 (24·1)	1·36	(0·40, 4·57)
Diagnosis				
Atrial fibrillation	7/17 (41·2)	5/12 (41·7)	1·25	(0·25, 6·22)
Sinus tachycardia	34/57 (59·6)	20/63 (31·7)	3·52	(1·57, 7·91)
Left ventricular ejection fraction (%)				
<50	13/27 (48·1)	9/25 (36·0)	1·66	(0·52, 5·29)
≥50	28/47 (59·6)	16/50 (32·0)	3·99	(1·59, 10·02)
Systolic blood pressure (mmHg)				
<120	18/39 (46·2)	16/44 (36·4)	2·22	(0·83, 5·92)
≥120	23/36 (63·9)	9/31 (29·0)	3·47	(1·14, 10·55)
Infection site				
Respiratory organ	11/22 (50·0)	5/23 (21·7)	16·51	(2·06, 132·09)
Other than respiratory organ	30/53 (56·6)	20/52 (38·5)	2·39	(1·04, 5·49)
Comorbid septic shock				
Yes	35/68 (51·5)	24/68 (35·3)	2·18	(1·04, 4·55)
No	6/7 (85·7)	1/7 (14·3)	Did not converge	Did not converge
Comorbid acute kidney injury				
Yes	24/43 (55·8)	17/54 (31·5)	2·71	(1·13, 6·51)
No	17/32 (53·1)	8/21 (38·1)	2·39	(0·65, 8·81)
Comorbid ARDS				
Yes	6/19 (31·6)	1/12 (8·3)	6·84	(0·61, 76·16)
No	35/56 (62·5)	24/63 (38·1)	3·11	(1·40, 6·92)
pH				
<7·35	11/28 (39·3)	4/19 (21·1)	1·81	(0·41, 7·92)
≥7·35	30/47 (63·8)	21/56 (37·5)	3·47	(1·46, 8·24)
Total SOFA score				
<10	20/32 (62·5)	10/33 (30·3)	5·67	(1·72, 18·66)
≥10	21/43 (48·8)	15/42 (35·7)	1·93	(0·75, 4·93)
APACHE II score				
<25	30/50 (60·0)	18/47 (38·3)	2·71	(1·10, 6·64)
≥25	11/25 (44·0)	7/28 (25·0)	2·82	(0·82, 9·67)
eGFR (mL/min/1·73 m^2)				
<30	12/26 (46·2)	12/38 (31·6)	2·15	(0·71, 6·50)
≥30	29/49 (59·2)	13/37 (35·1)	3·06	(1·17, 7·98)
pH <7·4 and HCO_3^- <24 mmol/L				
Yes	16/32 (50·0)	10/31 (32·3)	2·25	(0·75, 6·79)
No	25/43 (58·1)	15/44 (34·1)	3·44	(1·32, 8·92)
pH <7·4 and $PaCO_2$ >45 mmHg				
Yes	7/17 (41·2)	1/4 (25·0)	2·84	(0·21, 37·90)
No	34/58 (58·6)	24/71 (33·8)	3·16	(1·46, 6·82)

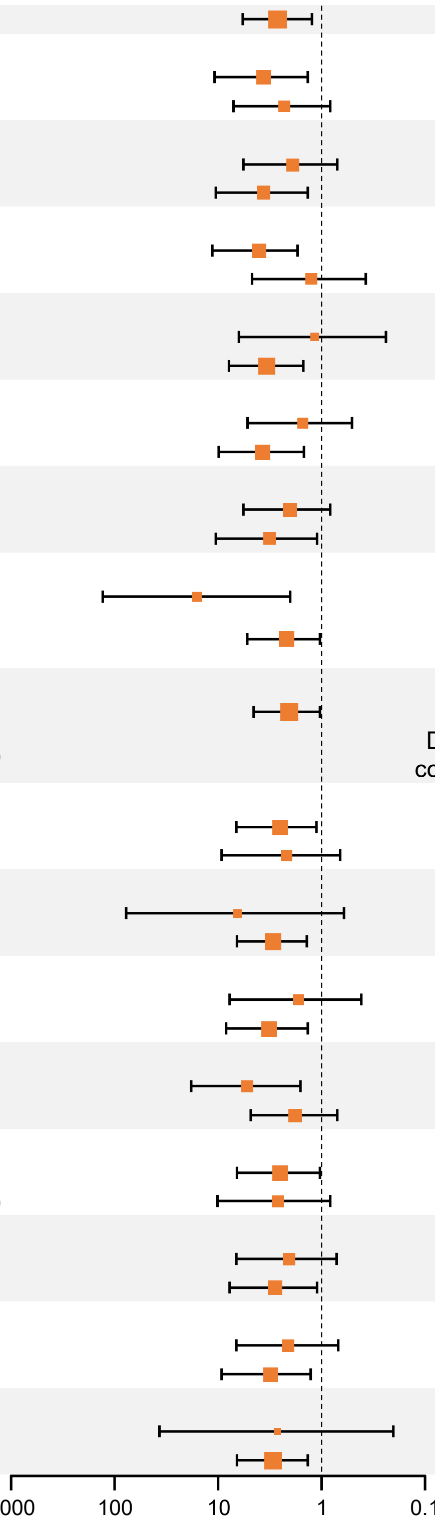

Favours landiolol Favours control

Odds ratio (95% CI) for the percentage of patients with HR of 60–94 beats/min at 24 h after randomisation

Figure S2. Post hoc, multivariate subgroup analysis of the percentage of patients who developed new arrhythmias by 168 h after randomisation using multivariate Cox proportional hazards model with adjustment for age and heart rate at baseline as covariates

The size of the markers represents the number of patients included in the subgroup. Hazard ratios are plotted using a log-scale. Results of univariate analyses by patient subgroups are shown in Fig. 3.

APACHE = Acute Physiology and Chronic Health Evaluation; ARDS = acute respiratory distress syndrome; beats/min = beats per minute; CI = confidence interval; eGFR = estimated glomerular filtration rate; NC = upper limit not calculable; SOFA = Sequential Organ Failure Assessment

Items	Landiolol group n/N (%)	Control group n/N (%)	Hazard ratio	(95% CI)
Overall	7/75 (9·3)	19/75 (25·3)	0·351	(0·147, 0·835)
Sex				
Male	4/52 (7·7)	9/38 (23·7)	0·308	(0·095, 1·003)
Female	3/23 (13·0)	10/37 (27·0)	0·447	(0·122, 1·632)
Age (years)				
<70	3/37 (8·1)	5/36 (13·9)	0·545	(0·129, 2·296)
≥70	4/38 (10·5)	14/39 (35·9)	0·264	(0·087, 0·802)
Heart rate (beats/min)				
<120	4/43 (9·3)	11/46 (23·9)	0·339	(0·108, 1·066)
≥120	3/29 (10·3)	8/29 (27·6)	0·365	(0·096, 1·380)
Diagnosis				
Atrial fibrillation	1/17 (5·9)	2/12 (16·7)	0·378	(0·034, 4·251)
Sinus tachycardia	6/57 (10·5)	17/63 (27·0)	0·390	(0·153, 0·993)
Left ventricular ejection fraction (%)				
<50	3/27 (11·1)	6/25 (24·0)	0·445	(0·111, 1·788)
≥50	4/47 (8·5)	13/50 (26·0)	0·303	(0·098, 0·932)
Systolic blood pressure (mmHg)				
<120	2/39 (5·1)	14/44 (31·8)	0·124	(0·027, 0·561)
≥120	5/36 (13·9)	5/31 (16·1)	0·997	(0·276, 3·607)
Infection site				
Respiratory organ	2/22 (9·1)	8/23 (34·8)	0·188	(0·040, 0·893)
Other than respiratory organ	5/53 (9·4)	11/52 (21·2)	0·486	(0·168, 1·400)
Comorbid septic shock				
Yes	7/68 (10·3)	18/68 (26·5)	0·378	(0·158, 0·905)
No	0/7 (0·0)	1/7 (14·3)	0·000	(0·000, NC)
Comorbid acute kidney injury				
Yes	3/43 (7·0)	14/54 (25·9)	0·272	(0·078, 0·949)
No	4/32 (12·5)	5/21 (23·8)	0·503	(0·127, 1·990)
Comorbid ARDS				
Yes	1/19 (5·3)	5/12 (41·7)	0·128	(0·015, 1·109)
No	6/56 (10·7)	14/63 (22·2)	0·446	(0·171, 1·162)
pH				
<7·35	3/28 (10·7)	5/19 (26·3)	0·382	(0·089, 1·647)
≥7·35	4/47 (8·5)	14/56 (25·0)	0·340	(0·112, 1·035)
Total SOFA score				
<10	2/32 (6·3)	7/33 (21·2)	0·236	(0·048, 1·153)
≥10	5/43 (11·6)	12/42 (28·6)	0·419	(0·146, 1·201)
APACHE II score				
<25	5/50 (10·0)	12/47 (25·5)	0·372	(0·131, 1·057)
≥25	2/25 (8·0)	7/28 (25·0)	0·280	(0·058, 1·361)
eGFR (mL/min/1·73 m^2)				
<30	3/26 (11·5)	14/38 (36·8)	0·309	(0·089, 1·078)
≥30	4/49 (8·2)	5/37 (13·5)	0·555	(0·147, 2·092)
pH <7·4 and HCO_3^- <24 mmol/L				
Yes	3/32 (9·4)	9/31 (29·0)	0·344	(0·093, 1·273)
No	4/43 (9·3)	10/44 (22·7)	0·365	(0·114, 1·165)
pH <7·4 and $PaCO_2$ >45 mmHg				
Yes	1/17 (5·9)	1/4 (25·0)	0·145	(0·007, 2·832)
No	6/58 (10·3)	18/71 (25·4)	0·394	(0·156, 0·993)

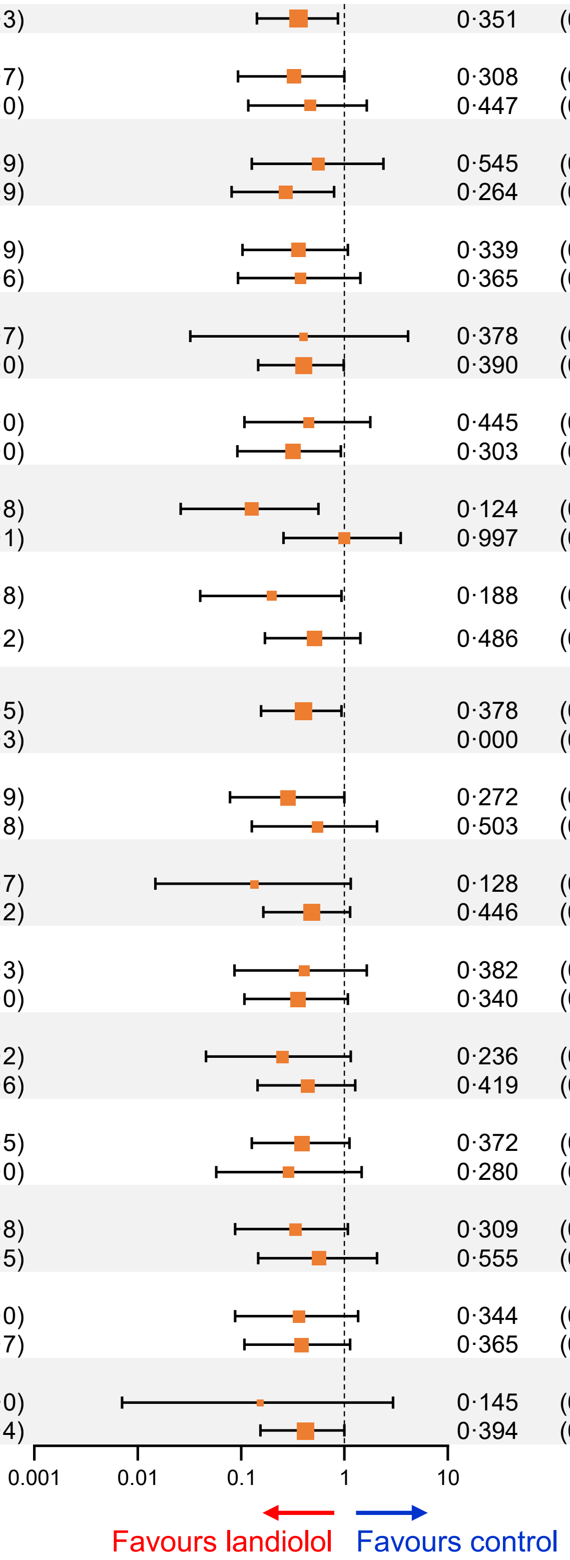

Hazard ratio (95% CI) for the percentage of patients who developed new arrhythmias by 168 h after randomisation

Figure S3. Post hoc, multivariate subgroup analysis of mortality by 28 days after randomisation using multivariate Cox proportional hazards model with adjustment for age and heart rate at baseline

The size of the markers represents the number of patients included in the subgroup. Hazard ratios are plotted using a log-scale. Results of univariate analyses by patient subgroups are shown in Fig. 4.

APACHE = Acute Physiology and Chronic Health Evaluation; ARDS = acute respiratory distress syndrome; beats/min = beats per minute; CI = confidence interval; eGFR = estimated glomerular filtration rate; NC = upper limit not calculable; SOFA = Sequential Organ Failure Assessment

Items	Landiolol group n/N (%)	Control group n/N (%)	Hazard ratio	(95% CI)
Overall	9/75 (12·0)	15/75 (20·0)	0·598	(0·261, 1·366)
Sex				
Male	6/52 (11·5)	10/38 (26·3)	0·442	(0·161, 1·218)
Female	3/23 (13·0)	5/37 (13·5)	0·901	(0·215, 3·781)
Age (years)				
<70	3/37 (8·1)	5/36 (13·9)	0·605	(0·144, 2·549)
≥70	6/38 (15·8)	10/39 (25·6)	0·595	(0·216, 1·639)
Heart rate (beats/min)				
<120	6/43 (14·0)	6/46 (13·0)	1·050	(0·338, 3·259)
≥120	3/29 (10·3)	9/29 (31·0)	0·345	(0·093, 1·279)
Diagnosis				
Atrial fibrillation	1/17 (5·9)	5/12 (41·7)	0·130	(0·015, 1·125)
Sinus tachycardia	8/57 (14·0)	10/63 (15·9)	0·914	(0·359, 2·325)
Left ventricular ejection fraction (%)				
<50	4/27 (14·8)	5/25 (20·0)	0·751	(0·201, 2·797)
≥50	5/47 (10·6)	10/50 (20·0)	0·514	(0·175, 1·504)
Systolic blood pressure (mmHg)				
<120	5/39 (12·8)	10/44 (22·7)	0·500	(0·164, 1·528)
≥120	4/36 (11·1)	5/31 (16·1)	0·670	(0·163, 2·755)
Infection site				
Respiratory organ	3/22 (13·6)	10/23 (43·5)	0·220	(0·059, 0·824)
Other than respiratory organ	6/53 (11·3)	5/52 (9·6)	1·392	(0·424, 4·567)
Comorbid septic shock				
Yes	8/68 (11·8)	13/68 (19·1)	0·631	(0·261, 1·522)
No	1/7 (14·3)	2/7 (28·6)	0·263	(0·018, 3·761)
Comorbid acute kidney injury				
Yes	7/43 (16·3)	10/54 (18·5)	0·975	(0·370, 2·566)
No	2/32 (6·3)	5/21 (23·8)	0·303	(0·056, 1·645)
Comorbid ARDS				
Yes	4/19 (21·1)	6/12 (50·0)	0·401	(0·107, 1·504)
No	5/56 (8·9)	9/63 (14·3)	0·607	(0·203, 1·813)
pH				
<7·35	6/28 (21·4)	7/19 (36·8)	0·503	(0·162, 1·559)
≥7·35	3/47 (6·4)	8/56 (14·3)	0·445	(0·118, 1·681)
Total SOFA score				
<10	0/32 (0·0)	4/33 (12·1)	0·000	(0·000, NC)
≥10	9/43 (20·9)	11/42 (26·2)	0·828	(0·340, 2·019)
APACHE II score				
<25	5/50 (10·0)	7/47 (14·9)	0·695	(0·220, 2·191)
≥25	4/25 (16·0)	8/28 (28·6)	0·508	(0·152, 1·701)
eGFR (mL/min/1·73 m^2)				
<30	5/26 (19·2)	9/38 (23·7)	0·860	(0·288, 2·569)
≥30	4/49 (8·2)	6/37 (16·2)	0·488	(0·138, 1·730)
pH <7·4 and HCO_3^- <24 mmol/L				
Yes	6/32 (18·8)	7/31 (22·6)	0·897	(0·301, 2·673)
No	3/43 (7·0)	8/44 (18·2)	0·342	(0·091, 1·288)
pH <7·4 and $PaCO_2$ >45 mmHg				
Yes	4/17 (23·5)	2/4 (50·0)	0·447	(0·077, 2·578)
No	5/58 (8·6)	13/71 (18·3)	0·471	(0·168, 1·320)

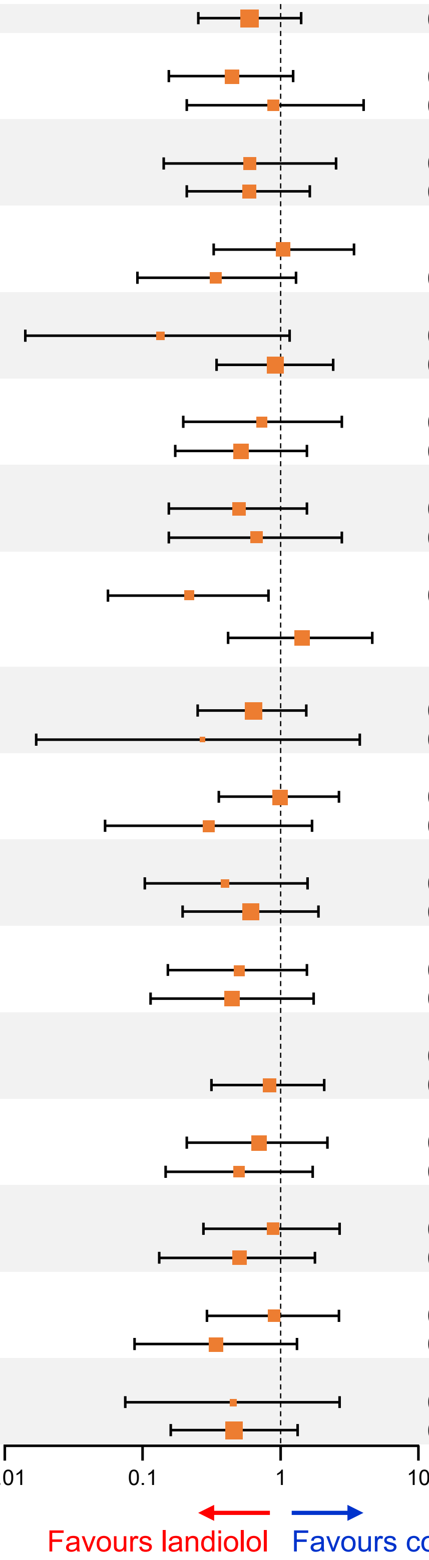

Hazard ratio (95% CI) for mortality by 28 days after randomisation

Figure S4. Post hoc, multivariate subgroup analysis of the percentage of patients with any adverse events using multivariate logistic regression with adjustment for age and heart rate at baseline

The size of the markers represents the number of patients included in the subgroup. Odds ratios are plotted using a log-scale. Results of univariate analyses by patient subgroups are shown in Fig. 5.

APACHE = Acute Physiology and Chronic Health Evaluation; ARDS = acute respiratory distress syndrome; beats/min = beats per minute; CI = confidence interval; eGFR = estimated glomerular filtration rate; SOFA = Sequential Organ Failure Assessment

Items	Landiolol group n/N (%)	Control group n/N (%)	Odds ratio	(95% CI)
Overall	49/77 (63·6)	44/74 (59·5)	1·20	(0·62, 2·32)
Sex				
Male	32/52 (61·5)	27/38 (71·1)	0·64	(0·26, 1·60)
Female	17/25 (68·0)	17/36 (47·2)	2·86	(0·92, 8·89)
Age (years)				
<70	25/38 (65·8)	22/35 (62·9)	1·18	(0·45, 3·12)
≥70	24/39 (61·5)	22/39 (56·4)	1·23	(0·50, 3·05)
Heart rate (beats/min)				
<120	29/43 (67·4)	27/46 (58·7)	1·48	(0·62, 3·54)
≥120	20/31 (64·5)	17/28 (60·7)	1·16	(0·40, 3·37)
Diagnosis				
Atrial fibrillation	8/17 (47·1)	6/12 (50·0)	0·87	(0·19, 3·97)
Sinus tachycardia	41/59 (69·5)	38/62 (61·3)	1·43	(0·67, 3·05)
Left ventricular ejection fraction (%)				
<50	19/28 (67·9)	17/25 (68·0)	1·00	(0·31, 3·25)
≥50	29/48 (60·4)	27/49 (55·1)	1·27	(0·56, 2·86)
Systolic blood pressure (mmHg)				
<120	26/40 (65·0)	26/44 (59·1)	1·51	(0·59, 3·87)
≥120	23/37 (62·2)	18/30 (60·0)	1·22	(0·44, 3·38)
Infection site				
Respiratory organ	15/22 (68·2)	15/23 (65·2)	1·12	(0·31, 4·03)
Other than respiratory organ	34/55 (61·8)	29/51 (56·9)	1·17	(0·53, 2·57)
Comorbid septic shock				
Yes	45/70 (64·3)	39/67 (58·2)	1·28	(0·64, 2·56)
No	4/7 (57·1)	5/7 (71·4)	0·49	(0·03, 7·21)
Comorbid acute kidney injury				
Yes	31/45 (68·9)	31/53 (58·5)	1·54	(0·67, 3·58)
No	18/32 (56·3)	13/21 (61·9)	0·89	(0·28, 2·85)
Comorbid ARDS				
Yes	10/19 (52·6)	11/12 (91·7)	0·09	(0·01, 0·92)
No	39/58 (67·2)	33/62 (53·2)	1·81	(0·86, 3·81)
pH				
<7·35	19/29 (65·5)	10/18 (55·6)	1·68	(0·49, 5·81)
≥7·35	30/48 (62·5)	34/56 (60·7)	1·09	(0·49, 2·43)
Total SOFA score				
<10	23/34 (67·6)	19/32 (59·4)	1·42	(0·50, 4·06)
≥10	26/43 (60·5)	25/42 (59·5)	0·91	(0·37, 2·25)
APACHE II score				
<25	32/52 (61·5)	28/46 (60·9)	1·03	(0·45, 2·33)
≥25	17/25 (68·0)	16/28 (57·1)	1·85	(0·57, 6·04)
eGFR (mL/min/1·73 m^2)				
<30	19/27 (70·4)	24/38 (63·2)	1·35	(0·46, 3·94)
≥30	30/50 (60·0)	20/36 (55·6)	1·22	(0·51, 2·91)
pH <7·4 and HCO_3^- <24 mmol/L				
Yes	24/33 (72·7)	19/30 (63·3)	1·52	(0·52, 4·44)
No	25/44 (56·8)	25/44 (56·8)	1·02	(0·44, 2·39)
pH <7·4 and $PaCO_2$ >45 mmHg				
Yes	9/17 (52·9)	2/4 (50·0)	1·09	(0·12, 10·14)
No	40/60 (66·7)	42/70 (60·0)	1·34	(0·65, 2·75)

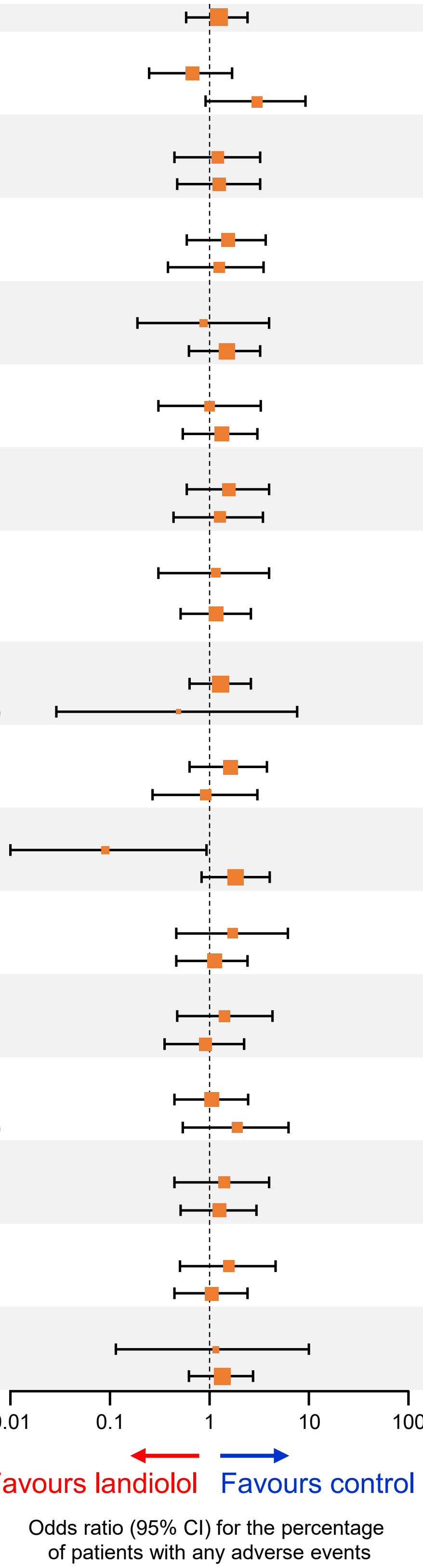

Supplement: Supplementary file 1 [file mmc1.pdf]
