# Supplementary material for: Impact of patient characteristics on the efficacy and safety of landiolol in patients with sepsis-related tachyarrhythmia: Subanalysis of the J-Land 3S randomised controlled study
Source: eClinicalMedicine. 2020 Oct 13;28:100571. doi: 10.1016/j.eclinm.2020.100571 (PMC7700908; doi:10.1016/j.eclinm.2020.100571)
Supplement: Supplementary file 2 [file mmc2.docx]

CONSORT 2010 checklist of information to include when reporting a randomised trial*


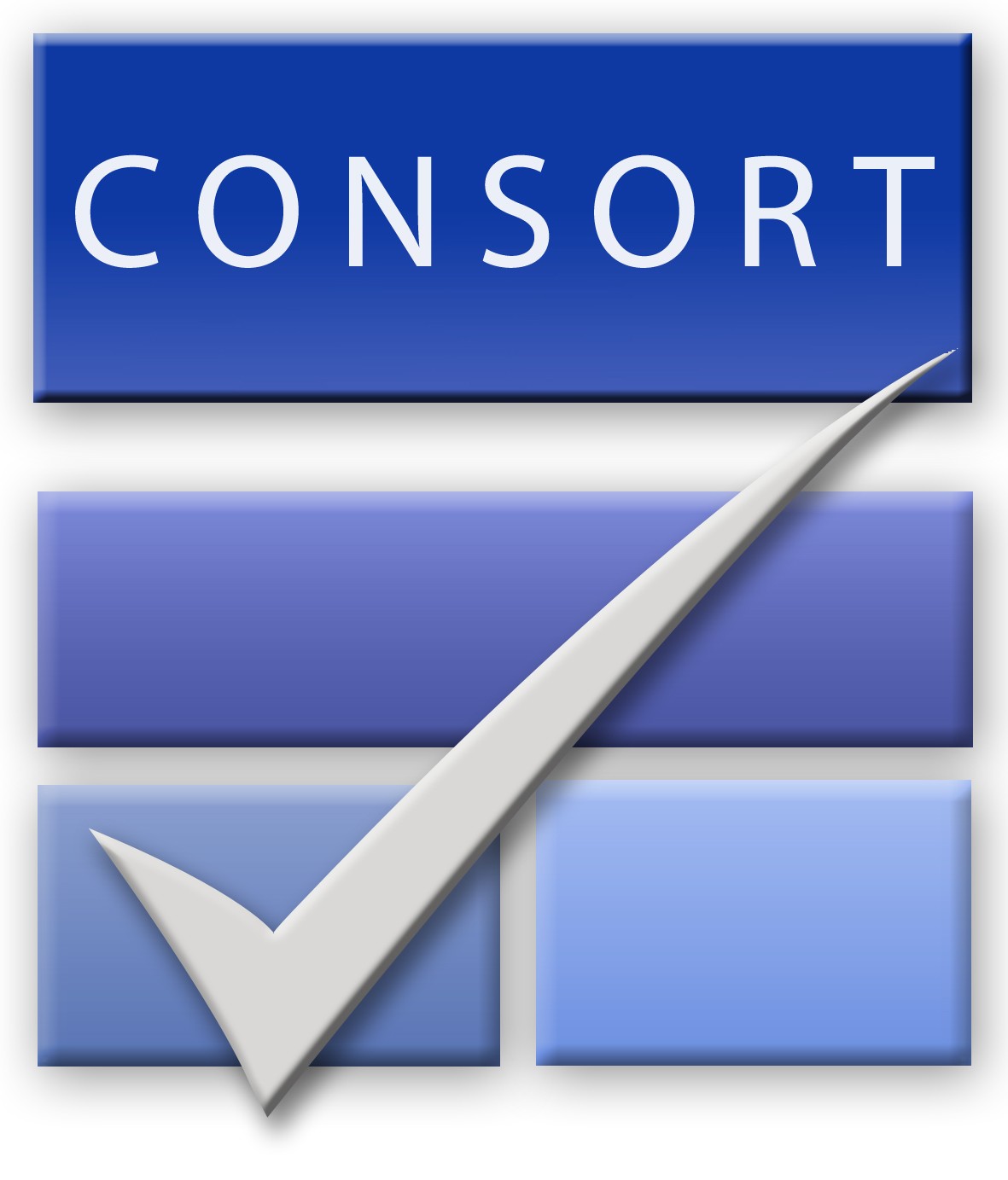


| Section/Topic | Item No | Checklist item | Reported on page No |
| --- | --- | --- | --- |
| Title and abstract | | | |
|  | 1a | Identification as a randomised trial in the title | 1 |
|  | 1b | Structured summary of trial design, methods, results, and conclusions (for specific guidance see CONSORT for abstracts) | 3-4 |
| Introduction | | | |
| Background and objectives | 2a | Scientific background and explanation of rationale | 5-6 |
|  | 2b | Specific objectives or hypotheses | 6 |
| Methods | | | |
| Trial design | 3a | Description of trial design (such as parallel, factorial) including allocation ratio | Prior article |
|  | 3b | Important changes to methods after trial commencement (such as eligibility criteria), with reasons | Prior article |
| Participants | 4a | Eligibility criteria for participants | 7 |
|  | 4b | Settings and locations where the data were collected | Prior article |
| Interventions | 5 | The interventions for each group with sufficient details to allow replication, including how and when they were actually administered | 8  Prior article |
| Outcomes | 6a | Completely defined pre-specified primary and secondary outcome measures, including how and when they were assessed | 8-9  Prior article |
|  | 6b | Any changes to trial outcomes after the trial commenced, with reasons | Prior article |
| Sample size | 7a | How sample size was determined | Prior article |
|  | 7b | When applicable, explanation of any interim analyses and stopping guidelines | Prior article |
| Randomisation: |  |  |  |
| Sequence generation | 8a | Method used to generate the random allocation sequence | Prior article |
|  | 8b | Type of randomisation; details of any restriction (such as blocking and block size) | Prior article |
| Allocation concealment mechanism | 9 | Mechanism used to implement the random allocation sequence (such as sequentially numbered containers), describing any steps taken to conceal the sequence until interventions were assigned | Prior article |
| Implementation | 10 | Who generated the random allocation sequence, who enrolled participants, and who assigned participants to interventions | Prior article |
| Blinding | 11a | If done, who was blinded after assignment to interventions (for example, participants, care providers, those assessing outcomes) and how | Prior article |
|  | 11b | If relevant, description of the similarity of interventions | Prior article |
| Statistical methods | 12a | Statistical methods used to compare groups for primary and secondary outcomes | Prior article |
|  | 12b | Methods for additional analyses, such as subgroup analyses and adjusted analyses | 8-10 |
| Results | | | |
| Participant flow (a diagram is strongly recommended) | 13a | For each group, the numbers of participants who were randomly assigned, received intended treatment, and were analysed for the primary outcome | 11, Fig 1  Prior article |
|  | 13b | For each group, losses and exclusions after randomisation, together with reasons | Prior article |
| Recruitment | 14a | Dates defining the periods of recruitment and follow-up | Prior article |
|  | 14b | Why the trial ended or was stopped | Prior article |
| Baseline data | 15 | A table showing baseline demographic and clinical characteristics for each group | Table 1  Prior article |
| Numbers analysed | 16 | For each group, number of participants (denominator) included in each analysis and whether the analysis was by original assigned groups | Figs 2-5, S1-S4 |
| Outcomes and estimation | 17a | For each primary and secondary outcome, results for each group, and the estimated effect size and its precision (such as 95% confidence interval) | Prior article |
|  | 17b | For binary outcomes, presentation of both absolute and relative effect sizes is recommended | N/A |
| Ancillary analyses | 18 | Results of any other analyses performed, including subgroup analyses and adjusted analyses, distinguishing pre-specified from exploratory | 11-14, Figs 2-5, S1-S4 |
| Harms | 19 | All important harms or unintended effects in each group (for specific guidance see CONSORT for harms) | 13  Prior article |
| Discussion | | | |
| Limitations | 20 | Trial limitations, addressing sources of potential bias, imprecision, and, if relevant, multiplicity of analyses | 20 |
| Generalisability | 21 | Generalisability (external validity, applicability) of the trial findings | 15-20 |
| Interpretation | 22 | Interpretation consistent with results, balancing benefits and harms, and considering other relevant evidence | 15-20 |
| Other information | | |  |
| Registration | 23 | Registration number and name of trial registry |  |
| Protocol | 24 | Where the full trial protocol can be accessed, if available | 7  Prior article |
| Funding | 25 | Sources of funding and other support (such as supply of drugs), role of funders | 4, 10, 22 |

**Please refer to our prior report for more information about the study, including information on items not reported in the present article.**

Kakihana Y, Nishida O, Taniguchi T, Okajima M, Morimatsu H, Ogura H, et al. Efficacy and safety of landiolol, an ultra-short-acting β1-selective antagonist, for treatment of sepsis-related tachyarrhythmia (J-Land 3S): a multicentre, open-label, randomised controlled trial. *Lancet Respir Med* 2020; 8: 863–72.

*We strongly recommend reading this statement in conjunction with the CONSORT 2010 Explanation and Elaboration for important clarifications on all the items. If relevant, we also recommend reading CONSORT extensions for cluster randomised trials, non-inferiority and equivalence trials, non-pharmacological treatments, herbal interventions, and pragmatic trials. Additional extensions are forthcoming: for those and for up to date references relevant to this checklist, see [www.consort-statement.org](http://www.consort-statement.org).
